# Differences between predictive factors for early neurological deterioration due to hemorrhagic and ischemic insults following intravenous recombinant tissue plasminogen activator

**DOI:** 10.1007/s11239-019-02015-4

**Published:** 2019-12-17

**Authors:** Koji Tanaka, Shoji Matsumoto, Konosuke Furuta, Takeshi Yamada, Sukehisa Nagano, Kei-ichiro Takase, Taketo Hatano, Ryo Yamasaki, Jun-ichi Kira

**Affiliations:** 1 Department of Neurology, Neurological Institute, Graduate School of Medical Sciences, Kyushu University, 3-1-1 Maidashi, Higashi-ku, Fukuoka, 812-8582 Japan; 2 Department of Neurology, Kokura Memorial Hospital, Kitakyushu, Japan; 3 Department of Comprehensive Strokology, Fujita Health University School of Medicine, Toyoake, Japan; 4 Department of Neurology, Saiseikai Fukuoka General Hospital, Fukuoka, Japan; 5 Department of Neurology, Fukuoka City Hospital, Fukuoka, Japan; 6 Department of Neurology, Iizuka Hospital, Iizuka, Japan; 7 Department of Neurosurgery, Kokura Memorial Hospital, Kitakyushu, Japan

**Keywords:** Early neurological deterioration, Risk factor, Thrombolysis, Symptomatic intracranial hemorrhage, Ischemic stroke

## Abstract

Early neurological deterioration (END) following intravenous recombinant tissue plasminogen activator (rt-PA) treatment is a serious clinical event that can be caused by hemorrhagic or ischemic insult. We investigated the differences in predictive factors for END due to hemorrhagic and END due to ischemic insults. Consecutive patients from four hospitals who received 0.6 mg/kg intravenous rt-PA for acute ischemic stroke were retrospectively recruited. END was defined as a National Institutes of Health Stroke Scale (NIHSS) score ≥ 4 points within 24 h compared with baseline. END was classified into those due to hemorrhagic (END_h_) or ischemic (END_i_) insult based on computed tomography (CT) or magnetic resonance imaging. Risk factors associated with END_h_ and END_i_ were investigated by comparison with non-END cases. A total of 744 patients (452 men, median 75 years old) were included. END was observed in 79 patients (10.6%), including 22 END_h_ (3.0%) and 57 END_i_ (7.7%), which occurred within a median of 7 h after treatment. Multivariate analyses showed that higher pretreatment NIHSS score (odds ratio [OR] 1.06, 95% confidence interval [CI] 1.00–1.13) and pretreatment with antiplatelets (OR 2.84, 95% CI 1.08–7.72) were associated with END_h_. Extensive early ischemic change (Alberta Stroke Program Early CT Score ≤ 7 on CT or ≤ 6 on diffusion-weighted imaging; OR 2.80, 95% CI 1.36–5.64) and large artery occlusions (OR 3.09, 95% CI 1.53–6.57) were associated with END_i_. Distinct factors were predictive for the END subtypes. These findings could help develop preventative measures for END in patients with the identified risk factors.

## Highlights

- Early neurological deterioration (END) following intravenous recombinant tissue plasminogen activator (rt-PA) for acute ischemic stroke is a serious clinical event; nevertheless, its etiology has not been well defined.
- Risk factors associated with END following intravenous rt-PA due to hemorrhagic (END_h_) or ischemic insults (END_i_) were investigated by comparing with non-END cases.
- Severe stroke symptoms and pretreatment with antiplatelets were associated with END_h_, and large-sized infarcts and large artery occlusions were associated with END_i_.
- Identification of these differences in predictive factors for END subtypes could inform the development of preventative measures for END following intravenous rt-PA.

## Introduction

Intravenous recombinant tissue plasminogen activator (rt-PA) is an effective treatment for acute ischemic stroke. However, early neurological deterioration (END), classically defined as any ≥ 4 points increase on the National Institutes of Health Stroke Scale (NIHSS) score within 24 h compared with baseline [1], occurs in more than 10% of patients who received intravenous rt-PA [2]. END leads to high mortality and poor functional outcomes [2, 3]. The primary causes of END have been reported to be symptomatic intracranial hemorrhage (sICH), malignant edema [2, 4], and early recurrent ischemic stroke (ERIS), the occurrence of new neurological symptoms involving initially unaffected vascular territories, and evidence of corresponding ischemic lesions [5, 6]. Other reported cases exhibit unexplained neurological deterioration; this is presumably caused by an infarct growth beyond the initial penumbra, which is often referred to as ‘stroke progression’ [7–9]. Previous studies have demonstrated that several clinical and radiological factors on admission and/or at 24 h were associated with END [1, 9–15]. While the detailed cause for deterioration has rarely been identified, there might be differences between the risk factors for END due to ischemic insults and those for END due to hemorrhagic insults. Indeed, a post hoc analysis of data from one randomized clinical trial showed a significant association between early addition of aspirin after intravenous rt-PA and END due to sICH but not due to cerebral ischemia [16]. Given this context, we hypothesized that there are different predictive factors for END due to ischemic insults and END due to hemorrhagic insults. Clarifying the risk factors for END subtypes might aid the development of preventative measures for END following intravenous rt-PA. Therefore, the aim of this study was to investigate differences in predictive factors for END due to hemorrhagic insults and END due to ischemic insults.

## Methods

### Subjects

We used data from a multicenter retrospective observational study, which has been previously described in detail [17]. Briefly, this study was conducted with patients from the stroke unit of four urban emergency hospitals (Saiseikai Fukuoka General Hospital, Fukuoka City Hospital, Iizuka Hospital, and Kokura Memorial Hospital). The subjects of this study were consecutive patients who received intravenous rt-PA for acute ischemic stroke between October 1st, 2005 and December 31st, 2015. All patients received intravenous administration of 0.6 mg/kg alteplase in accordance with the Japanese guidelines [18]. This study was approved by the ethics committees of Kyushu University Hospital (29-111) and those of each of the four facilities. Written informed consent was waived because of the retrospective study design.

The following clinical information was systematically collected from medical records: age, sex, vascular risk factors (hypertension, diabetes mellitus, and dyslipidemia), atrial fibrillation (AF), previous history of stroke, and pretreatment with antiplatelets and anticoagulants. Severity of stroke symptoms was assessed by the NIHSS score, which was obtained before the administration of rt-PA. Computed tomography (CT) and/or magnetic resonance imaging (MRI) including diffusion-weighted imaging (DWI) were performed prior to administration of rt-PA for the assessment of early ischemic change (EIC). At least two experienced physicians retrospectively evaluate EIC using the Alberta Stroke Program Early CT Score (ASPECTS) [19, 20] on CT and/or DWI in each facility, without using a central reading system. Extensive EIC was defined as an ASPECTS of ≤ 7 on CT or ≤ 6 on DWI. Arterial occlusion sites were assessed by MR angiography, carotid ultrasonography, and/or CT angiography. Large artery occlusions were defined as one or more occlusions of the internal carotid artery (ICA), proximal portion of the middle cerebral artery (MCA), and/or the basilar artery (BA) detected by any modality. The pretreatment systolic and diastolic blood pressure and glycemia, and onset-to-needle time were obtained from emergency medical charts. Endovascular therapy alongside thrombolysis was performed for eligible patients, including mechanical clot disruption and retrieval, and angioplasty with or without stenting.

### Definition of END and subtypes

**Fig. 1:**
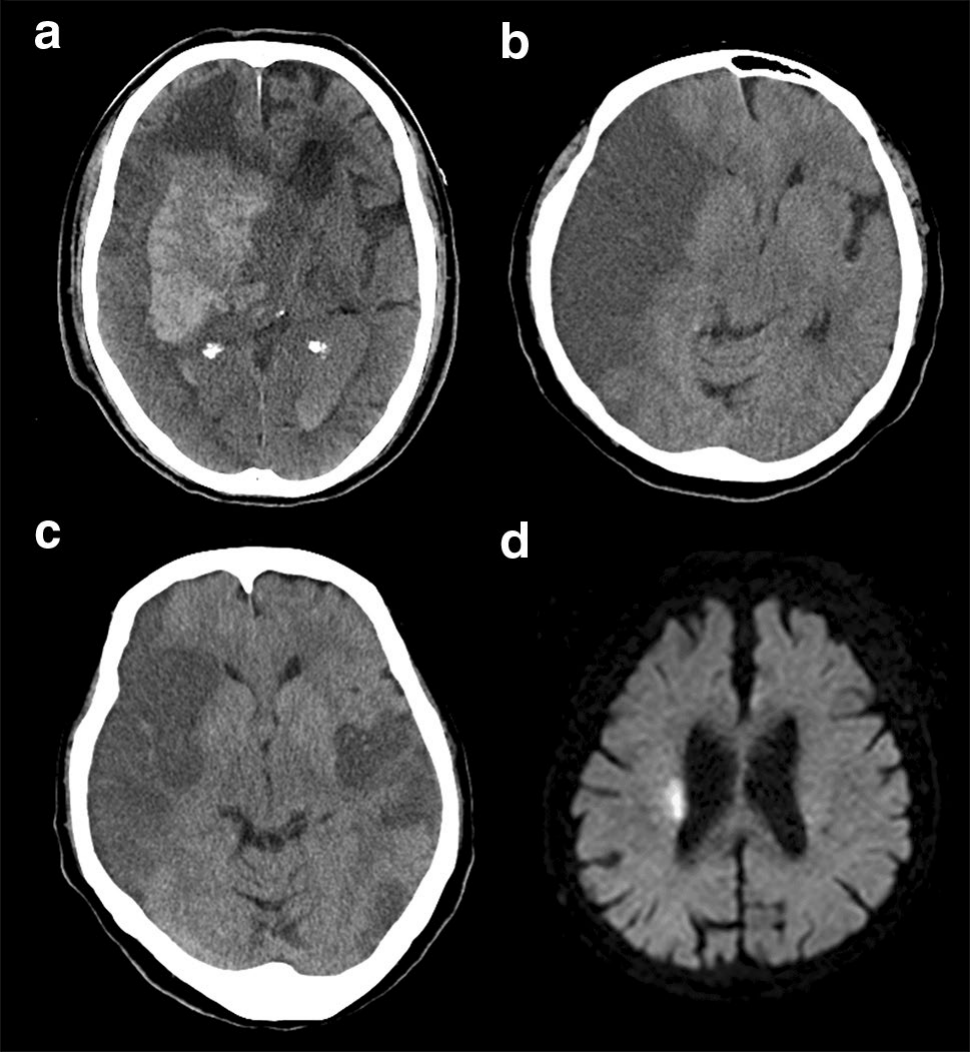
Representative brain imaging of patients with early neurological deterioration due to hemorrhagic (**a**) and ischemic (**b**–**d**) insults. **a** Axial computed tomography (CT) performed 8 h after thrombolysis showed an extended parenchymal hematoma of the right basal ganglia with additional blood in both lateral ventricles and hydrocephalus. **b** Axial CT performed 18 h after thrombolysis showed extensive brain edema and midline shift. **c** Axial CT performed 12 h after thrombolysis in a patient who became comatose during thrombolysis for ischemic stroke in the left middle cerebral artery (MCA) territory showed new acute ischemic lesions in the right MCA territory. **c** Diffusion-weighted imaging obtained 12 h after thrombolysis showed a hyperintense lesion in the territory of lenticulostriate arteries, which did not change much from baseline

After the treatment, patients’ symptoms were closely monitored. END was defined as a neurological deterioration with a ≥ 4 points increase on the NIHSS score compared with baseline within 24 h after the administration of rt-PA. The time of END onset was collected from medical records. Brain imaging was principally performed at the time of deterioration and/or 24 h after the treatment. Each END case was retrospectively reviewed and classified into those due to hemorrhagic (END_h_) or ischemic (END_i_) insults as follows: END_h_ is an END presumably caused by a parenchymal intracerebral hematoma, which refers to a hematoma with a mass effect occupying 30% or more of the infarct in the ischemic region (Fig. 1a) or subarachnoid hemorrhage; END_i_ is an END other than END_h_, and which includes malignant edema, ERIS, and unexplained neurological deterioration (Fig. 1b–d). Patients whose symptoms deteriorated as a result of other clinically apparent causes were excluded.

### Statistical analysis

All statistical analyses were performed using JMP statistical software version 9.0 (SAS Institute Inc., Cary, NC, USA). Data are expressed as medians and interquartile ranges for continuous variables and counts and percentages for categorical variables. Clinical characteristics were compared using the Chi squared test, Fisher’s exact test, or Wilcoxon rank sum test as appropriate. We then performed multivariate logistic regression analyses including variables that were significantly associated with END_h_ and END_i_ in the univariate analysis to identify factors associated with both END_h_ and END_i_. A p-value of < 0.05 was considered statistically significant.

## Results

**Fig. 2:**
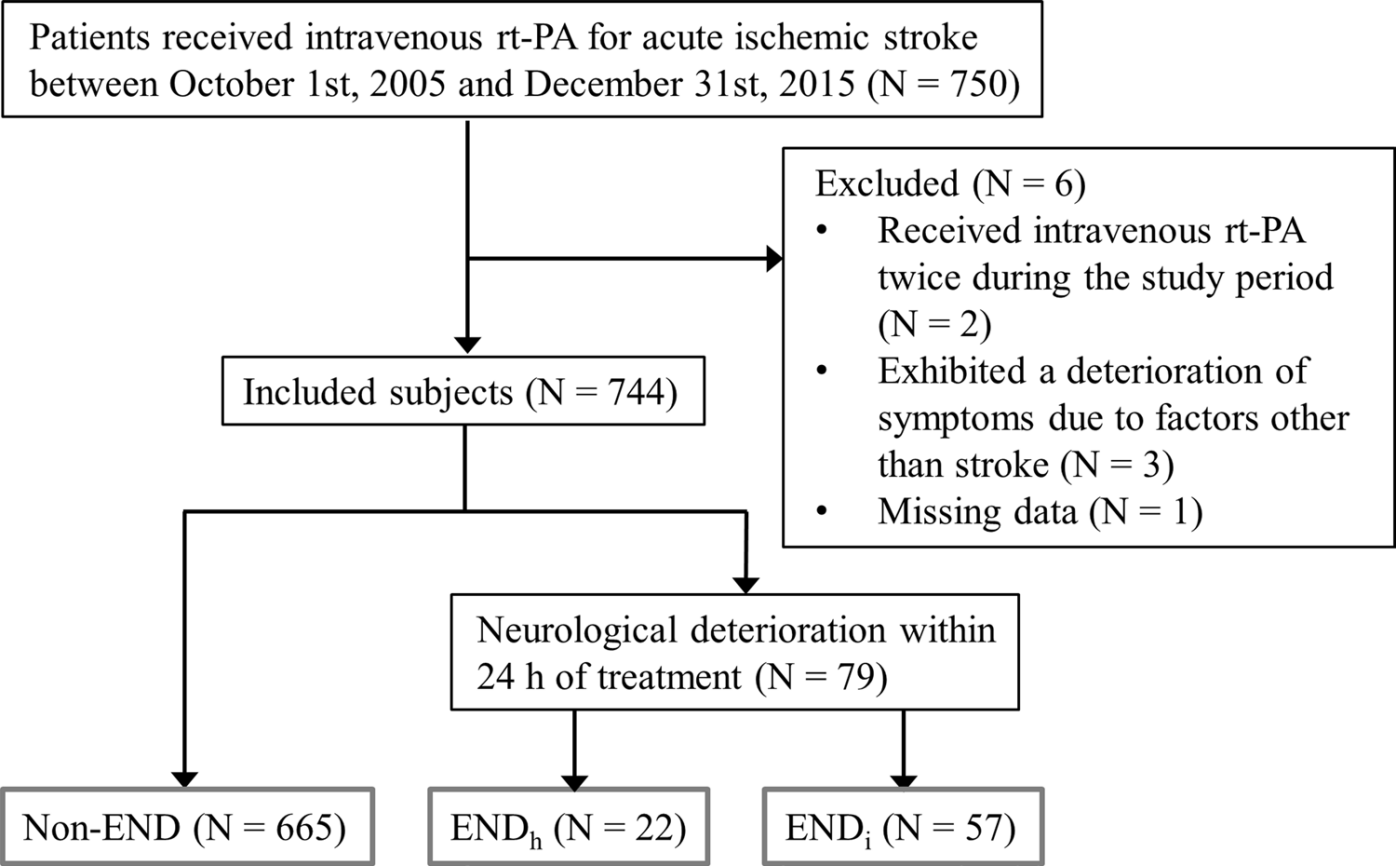
Flowchart of patient selection. END, early neurological deterioration; END_h_, END due to hemorrhagic insult; END_i_, END due to ischemic insult

A total 750 patients that had received intravenous rt-PA were registered during the study period. A flowchart of patient selection is shown in Fig. 2. Among these, 6 patients were excluded because they had received intravenous rt-PA twice during the study period (n = 2), exhibited a deterioration of symptoms due to factors other than stroke (cardiac arrest due to ventricular arrhythmia [n = 2] and intubation due to heart failure [n = 1]), or missing data (n = 1). Finally, 744 patients (452 men, median 75 years old) were included in the analysis. Antiplatelets were prescribed to 204 patients (27.4%) prior to stroke, including aspirin (n = 167), clopidogrel (n = 45), and/or others (n = 25). Thirty-three patients (4.4%) received dual antiplatelet therapy. Anticoagulants were prescribed to 121 patients (16.3%), including warfarin (n = 109), direct oral anticoagulants (n = 8), or unfractionated heparin (n = 4). Before thrombolysis, CT was performed in 651 patients (87.5%) and 54 patients (8.3%) were classified as having an ASPECTS ≤ 7; MRI was performed in 617 patients (82.9%) and 79 patients (12.8%) were classified as having an ASPECTS ≤ 6 on DWI. As a result, 99 out of the 744 patients (13.3%) were classified as having extensive EIC. Large artery occlusions were seen in 306 patients (47.8%) out of 640 patients who were evaluated for arterial occlusion sites before thrombolysis, and these included ICA (n = 102), proximal portion of MCA (n = 165), and BA (n = 39). Endovascular therapy alongside thrombolysis was received by 82 patients (26.8%) with large artery occlusions. END was seen in 79 patients (10.6%) including 22 END_h_ (3.0%) and 57 END_i_ (7.7%). END occurred a median of 7 h after the initiation of treatment (8 [1.75–17.25] hours in END_h_ and 4 [1.0–13.5] hours in END_i_, p = 0.147), and most of them occurred within the first 2 h.

Univariate analyses of clinical characteristics between non-END patients and patients with END_h_ and END_i_ are shown in Table 1. Compared with non-END patients, the extensive EIC and large artery occlusions were more common in patients with both END subtypes; the pretreatment NIHSS score was higher (20.5 [16–26] vs. 14 [8–20], p = 0.001) and pretreatment with antiplatelets was more common (50.0% vs. 27.7%, p = 0.022) in patients with END_h_, and AF was more common in patients with END_i_ (64.9% vs. 49.3%, p = 0.027). The multivariate analyses revealed that a higher pretreatment NIHSS score (odds ratio [OR] 1.06, 95% confidence interval [CI] 1.00–1.13) and pretreatment with antiplatelets (OR 2.84, 95% CI 1.08–7.72) were associated with END_h_. Eleven patients with antiplatelet pretreatment developed END_h_, and all of them had taken aspirin (9 patients with aspirin only, 1 with dual antiplatelet therapy, and 1 with aspirin and warfarin). Extensive EIC (OR 2.80, 95% CI 1.36–5.64) and large artery occlusions (OR 3.09, 95% CI 1.53–6.57) were associated with END_i_.

**Table 1:** Univariate analysis of clinical characteristics in patients with early neurological deterioration due to hemorrhagic and ischemic insults

	Total N = 744	Non-END N = 665	END_h_ N = 22	p-value	END_i_ N = 57	p-value
Sex, male	452 (60.8)	406 (61.1)	16 (72.7)	0.374	30 (52.6)	0.212
Age (years)	75 (66–82)	75 (66–82)	78 (67–87.25)	0.154	75 (64–81)	0.854
Hypertension	489 (65.7)	436 (65.6)	15 (68.2)	1.000	38 (66.7)	0.866
Dyslipidemia	199 (26.7)	183 (27.5)	5 (22.7)	0.809	11 (19.3)	0.179
Diabetes mellitus	170 (22.8)	147 (22.1)	8 (36.4)	0.123	15 (26.3)	0.465
Atrial fibrillation*	378 (50.8)	328 (49.3)	13 (59.1)	0.394	37 (64.9)	0.027
Previous history of stroke	124 (16.7)	112 (16.8)	3 (13.6)	1.000	9 (15.8)	1.000
Pretreatment with antiplatelets*	204 (27.4)	184 (27.7)	11 (50.0)	0.022†	9 (15.8)	0.061
Pretreatment with anticoagulants	121 (16.3)	110 (16.5)	4 (18.2)	0.773	7 (12.3)	0.460
Glycemia (mmol/L)	6.94 (6.00–8.47)	6.89 (5.94–8.39)	7.72 (5.56–9.42)	0.404	7.22 (6.22–10.09)	0.067
Systolic blood pressure (mmHg)	160 (141–180)	159 (141–180)	160 (149–173)	0.745	160 (138–183)	0.556
Diastolic blood pressure (mmHg)	85 (72–100)	85 (72–99)	85 (72–96)	0.938	89 (73–104)	0.471
Extensive EIC*	99 (13.3)	74 (11.1)	7 (31.8)	0.010	18 (31.6)	< 0.001†
Large artery occlusions (N = 640)*	306 (47.8)	256 (44.8)	13 (72.2)	0.029	37 (74.0)	< 0.001†
Pretreatment NIHSS score*	15 (8–20)	14 (8–20)	20.5 (16–26)	0.001†	16 (10–21.5)	0.061
Onset-to-needle time (min)	140 (110–174)	141 (112–175)	130 (99–162)	0.394	130 (101–162)	0.114
Endovascular therapy	122 (16.4)	107 (16.1)	2 (9.1)	0.556	13 (22.8)	0.196

Data are presented as N (%) or median (interquartile range)

*END* early neurological deterioration, *END*_*h*_ END due to hemorrhagic insult, *END*_*i*_ END due to ischemic insult, *EIC* early ischemic change, *NIHSS* National Institutes of Health Stroke Scale

*Included in the multivariate model

†Variables that maintain the level of significance in multivariate analysis

## Discussion

This study found that END occurred in approximately one-tenth of patients who received intravenous rt-PA for acute ischemic stroke. Among them, sICH accounted for one-third of the overall cause of END. This frequency and proportion of END was comparable to previous studies in which 0.9 mg/kg alteplase was administrated [1, 2, 7, 9, 13, 15]. The median time from administration of rt-PA to deterioration was 8 h for END_h_ and 4 h for END_i_, and mainly occurred within first 2 h. Similar results were reported in a recent study in which END occurred at a mean of 7.3 h after rt-PA administration in patients with sICH, and 4.8 h in those without [15]. Moreover, ERIS has been reported to occur during or shortly after rt-PA administration [5, 6]. These data indicate that END occurs early after the administration of rt-PA, regardless of hemorrhagic or ischemic insults.

This study identified some distinct factors that were predictive for END subtypes; severe stroke symptoms and pretreatment with antiplatelets were associated with END_h_, and extensive EIC and large artery occlusions were associated with END_i_. Similar to previous studies [21–23], a higher pretreatment NIHSS score was associated with END_h_. In the Japan Alteplase Clinical Trial [24], 5 of 6 patients who developed sICH had a pretreatment NIHSS score of ≥ 19. These results conflict with the reported association between END due to ischemia and lower baseline NIHSS scores, which were suggestive of good collateral flow at baseline [3, 7]. Imaging studies have demonstrated that lower residual cerebral blood flow and lower apparent diffusion coefficient value in ischemic lesions were predictive of sICH [25, 26]. Taken together, a higher NIHSS score rather than a lower ASPECTS might be suggestive of a greater depth of ischemia with irreversible tissue damage, which leads an increased risk of hemorrhage on reperfusion.

We found a significant association between pretreatment with antiplatelets and END_h_. Although the association between pretreatment with antiplatelets and overall END has not been reported in previous studies, this result was similar to that of early addition of aspirin after intravenous rt-PA and END due to sICH [16]. This may help to predict which patients are prone to END_h_ and may also inform early post-thrombolytic management (e.g. stricter blood pressure control and restricted use of nonsteroidal anti-inflammatory drugs within the initial 24 h).

An extensive EIC, defined as an ASPECTS of ≤ 7 on CT or ≤ 6 on DWI, represents a large-sized infarct or multiple acute cerebral infarcts. Krieger et al. [27] demonstrated that a hypodensity of > 50% of the MCA territory on initial CT predicts fatal brain swelling. An ASPECTS of ≤ 7 on CT has been associated with extensive EIC in the one-third MCA territory method [28]. Thus, extensive EIC representing a large-sized infarct in the MCA territory might be associated with a high risk for END due to brain edema. Multiple acute cerebral infarcts are another cause of the extensive EIC, and presumably derive from symptomatic ICA stenosis/occlusion or cardiac embolism [29], which are known predictors of END_i_ [3, 6, 9].

Large artery occlusions were surrogate markers for not only the penumbra, but also the oligemia. In a previous report from Seners et al. [7], alongside the proximal occlusion of large arteries, no-recanalization was a predictor for END due to causes other than sICH or malignant edema. The secondary hemodynamic or metabolic disruption of the oligemic tissue mainly due to the loss of collateral flow has been suggested to be one of the major mechanisms of unexplained neurological deterioration [7–9]. In this study, only 26.8% of patients with large artery occlusions underwent endovascular therapy adjacent to thrombolysis. This finding suggests that the reduction in the incidence of END_i_ is one of the therapeutic effects of the recent mechanical thrombectomy alongside thrombolysis in patients with acute ischemic stroke and large artery occlusions.

This study has several limitations. First, this study had a retrospective design with a limited number of patients and facilities, which could lead to some selection bias and statistical errors. Second, we did not study some pre- and post-treatment parameters that could potentially affect the incidence of END (e.g. time from taking antithrombotic agents to the treatment, blood pressure variability, no recanalization, and arterial reocclusion). Third, ischemic lesions in the posterior circulation might be overlooked by the ASPECTS. Fourth, the associations between END subtype and clinical outcome were unclear, because the 3-month modified Rankin Scale was not investigated in the present study. Finally, some END related to endovascular procedures might be included, because patients received endovascular treatment alongside thrombolysis were not excluded.

In conclusion, END occurred in approximately one-tenth of patients receiving intravenous rt-PA, and END_i_ was three times more common than END_h_. Distinct factors were associated with END subtypes. Our findings might inform the development of preventative measures for END following intravenous rt-PA in patients with these risk factors.
